# Being publicly diagnosed: A grounded theory study of Danish patients with tuberculosis

**DOI:** 10.3402/qhw.v9.23644

**Published:** 2014-04-23

**Authors:** Hanne Konradsen, Troels Lillebaek, Torgny Wilcke, Kirsten Lomborg

**Affiliations:** 1 Gentofte University Hospital, Hellerup, Denmark; 2 International Reference Laboratory of Mycobacteriology, Statens Serum Institut, Copenhagen, Denmark; 3 Department of Nursing Science, Aarhus University, Aarhus N, Denmark

**Keywords:** Tuberculosis, public health, grounded theory, interview, observation, health care, infectious disease, pulmonary, nursing

## Abstract

**Introduction:**

Tuberculosis (TB) is a disease which affects people worldwide, but there is knowledge lacking about patients’ experiences in low-prevalence and high-income countries.

**Aim:**

To provide a theoretical framework for the process of being diagnosed with tuberculosis in a Danish setting.

**Method:**

A grounded theory design with field studies and qualitative interviews, following the recommendations from Glaser and Strauss.

**Result:**

A process of being publicly diagnosed was identified, which developed during the patient's trajectory from being on the way to becoming a patient, becoming a patient with TB, and finally being in medical treatment. Before being diagnosed with TB, patients were weighing between biding their time and deciding to undergo an examination. Social pressure and feelings of social responsibility tended to affect the decision. Having undergone the examination(s), the patients were publicly diagnosed. Being publicly diagnosed meant changing social interactions and fighting to regain control.

**Conclusion:**

Findings offer new insight and an empirically derived basis for developing interventions aimed at reducing the burden of being diagnosed with tuberculosis and increasing the wellbeing of the patients.

Tuberculosis (TB) is a disease which affects people worldwide. Historically, *Mycobacterium tuberculosis* (MT) has caused more deaths than any other contagious microorganism. It is thought that about one-third of the world's population is latently infected, and the incidence of newly diagnosed patients was almost 9 million in 2011 (WHO, 2012). If patients do not complete a course of treatment, there is a risk of MT transmission, drug resistance, relapse, or death (Raviglione, Snider, & Kochi, 1995; Volmink & Garner, 2007). TB mortality is closely related to social inequality (Alvarez et al., 2011). From a social perspective, it is most urgent to address the enormous global inequalities in living conditions, wealth, and access to healthcare (Gandy & Zumla, 2002).

In Europe, it is estimated that more than 400,000 new cases of TB occur every year (Dara et al., 2012). The HIV epidemic, increased migration, and the partial collapse of healthcare provision in Eastern Europe, together with continued active MT transmission, cause TB to remain a health challenge (Lillebaek, Andersen, Seersholm, & Thomsen, 2012). In Denmark, there were 360 newly diagnosed TB patients in 2011 (WHO, 2012), and because the active transmission of MT is still not under control, these numbers are expected to increase in the future (Lillebaek et al., 2012). The group of patients with TB represents a mix of Danes and immigrants, but there is only limited MT transmission between the two groups (Kamper-Jorgensen et al., 2012). Because TB in Denmark is a notifiable contagious disease, treatment is free but any new patient must be reported to the medical officer of health and the National Health Surveillance, and an active search for the source of infection or potentially infected contacts must commence. This is done in order to find the source of infection and to find other potentially infected persons, and thus break the transmission chain. Most of our knowledge concerning TB-related healthcare seeking, adherence, and compliance is limited, and relates to countries with high TB prevalence and low incomes (Munro et al., 2007; National Collaborating Center for Chronic Conditions, 2006).

Treatment of TB requires taking multiple drugs for at least 6 months, and is considered unique, as medical treatment continues for a long time after clinical improvement in order to prevent relapse (Pozsik, 1993). Directly Observed Treatment (DOT) was first adopted in the 1960s and is now widely recommended for the control of TB, even though no significant difference has been found between DOT and self-administration in terms of cure or completion of treatment (Volmink & Garner, 2007). WHO has stated that “promoting adherence through a patient-centred approach is probably more effective in preventing treatment interruption than devoting resources to patients who default” (WHO, 2010). Several interacting factors affect adherence to TB medication, including structure, social background and context, health service, and personal factors. There is a general lack of knowledge concerning people who are living with TB and undergoing treatment (Munro et al., 2007) and to our knowledge, there are no systematic records of patients’ experiences and concerns in connection with being diagnosed with TB in a high-income, low-prevalence setting such as Denmark.

## Objectives

The aim of this qualitative study was to provide a theoretical framework for the process of being diagnosed with TB in a Danish setting.

## Method

A grounded theory design with field studies and qualitative interviews was chosen, with the aim of discovering the most significant empirically derived multivariate, and contextualized basic social process of being diagnosed with TB. We have followed the recommendations of Glaser and Strauss (Glaser & Strauss, 1968) and Glaser's elaboration of theoretical sensitivity (Glaser, 1978). Because the intention was to inform practice, a grounded theory approach was useful, because it explains, accounts for, and interprets “the variation in behaviour in the substantive area under study, most often hinged around processing a problem for the subjects” (Glaser, 1992).

### Sample and data analysis

Concurrent data collection and theoretical sampling took place at two different university hospitals in Denmark from October 2011 to April 2013. The data consisted of individual formal and non-formal interviews with patients, observations of interactions and conversations between nurses and patients, and observations of various nurses talking to relatives, colleagues, and other non-patients. Furthermore, observations were made of nurses involved in outreach work in shelters for the homeless and drug addicts, who were engaged in finding potential patients and encouraging them to have a sputum sample examined for MT.

The patients included had various trajectories in relation to the diagnosis. Some had for some time lived with unexplained symptoms, such as coughing, fever, or chest pain, and many had undergone a number of examinations and antibiotic treatments before the final TB diagnosis was made. Some had had the disease for a while without noticing it, and were diagnosed because they agreed to be examined as contacts to a person who had TB, or because they contributed a sputum sample during a screening programme. A total of 14 patients were individually interviewed. Patients were selected on the basis of having an appointment at the ward, at randomly selected days where the first author was present. The interviews took place at the hospital, in a quiet room outside the outpatient clinic. Each patient chose the time for the interview, and all wished it to follow one of their regular appointments in the outpatient clinic. Observations of interactions and conversations between nurses and patients took place on 8 randomly selected days, and each day involved between 5 and 7 h of observation. As a significant part of patients who are diagnosed with TB also struggle with substance abuse and other social problems, this limited the possibility of making appointments for formal interviews. Accordingly, many informal conversations were also undertaken with patients during the observations. Field notes and memos were taken during observations.

Initially, the data, consisting of field notes and transcripts from interviews and theoretical memos, were openly coded line-by-line. The codes were constantly compared back and forth, both within the individual interviews and across all data, in an attempt to answer the question “What is happening here?” In accordance with the principles of grounded theory, the research protocol was not viewed as a rigid template. Initially, the main focus was on the patients’ experiences of being treated for TB, but by being open-minded it became clear to the researchers that the main concern for the patients was being diagnosed with TB. Using the principles of theoretical sampling, this then became the focus. An example of theoretical sampling was the decision to collect data outside the hospitals, because initial analysis caused a shift from focus on treatment to focus on diagnosis. Analysis continued until theoretical subcategories and possible links between them emerged. Diagramming was used during this process, both to stimulate the theoretical attention and to consider links and relationships between concepts. This continued until the main concept (being publicly diagnosed) emerged and theoretical saturation was achieved. Afterwards, the main concern of the patients was subjected to further exploration, and the development of a theoretical model was confirmed.

### Validity

Some of the authors were experienced in caring for patients with TB and some not. This was seen as strengthening the validity of the study, because discussions in the analytic process thereby opened up for new perspectives, interpretations, and dimensions in the analysis by combining analytic questions from both an inside and outside position. In grounded theory four quality criteria are established; fit, relevance, modifiability, and workability (Glaser & Strauss, 1968). From a scientific realist perspective “fit” means correspondence between the theory and the empirical study, and “fit” is therefore closely related to study validity (Lomborg & Kirkevold, 2003). The researchers’ constant comparative method followed the principles of grounded theory, starting with an inductive, open phase and moving to a deductive phase once the core category was defined. This analytic process ensured correspondence between the raw data and the final theoretical construct. We therefore believe the theory is valid even though always contemporary and modifiable along with contextual changes e.g., in TB treatment and societal preventive TB control. We discussed findings with other healthcare professionals, in order to ensure relevance. All together these procedures should warrant the quality of the study.

### Ethical considerations

The Ethics Committee of Copenhagen (number H-4-2013-088) was queried for ethical approval and the Danish Data Protection Agency. The managers of the two participating hospitals were given oral information about the study, and the nurses were given both oral and written information. None refused to participate. The patients were given oral information when they were approached for formal interviews, and were given written information about the study together with the assurance of anonymity, voluntary participation, and the right to withdraw their consent. When collecting data through participant observations, the healthcare professionals were repeatedly asked about the appropriateness of the researcher being present, and during informal conversations the researcher who performed the data collection presented herself as a researcher (Oeye, Bjelland, & Skorpen, 2007).

## Results

In this study we identified a process of *being publicly diagnosed*, which developed during the patient's trajectory from being on the way to becoming a patient, becoming a patient with TB, and finally being in medical treatment. Before being diagnosed with TB, patients who had symptoms related to TB, or who were offered voluntary examinations, were weighing between *biding their time* and *deciding to undergo an examination*. Social pressure and feelings of social responsibility tended to affect the decision. Having undergone the examination(s), the patients were *publicly diagnosed*. *Being publicly diagnosed* meant *changing social interactions* and *fighting to regain control*.

### On the way to becoming a patient

Before being diagnosed with TB, the patients went on with their daily lives. Two different kinds of events disrupted that life: either the patient began to experience physical symptoms which slowly increased in either severity or number, or the patient regularly visited places where healthcare professionals offered participation in TB screening projects.

The physical symptoms that suggested that something was wrong did not usually appear suddenly. Typically, the symptoms were at first expected to go away after a while. The increase in either the severity or number of symptoms demanded consideration of whether the symptoms were a sign of illness, prompting a weighing-up whether to go on biding one's time or deciding to undergo examination(s). One man, who smoked and was used to coughing a lot, especially in the mornings, put it this way:

I started coughing all day long and just thought I had caught a cold. Then it got worse and I just felt more and more tired. That's when I knew something was wrong. Looking back now, I know exactly when it began, but I didn't think it was anything special at the time. (Interview 2)

The weighing-up period between biding one's time and deciding to undergo an examination could go on for some time. Sitting in a free public clinic where anyone could come and deliver a sputum test, a man was observed standing outside on the pavement and waiting until the last moment to come in. “I have really had to pull myself together to come here,” he said when he reluctantly entered the clinic (observation day 5).

Patients without physical symptoms were faced with the same weighing-up process between deciding to undergo examination or biding their time, either because they were offered an examination because they were considered to belong to an at-risk population, or because they had had contact with a person who had TB.

Social pressure or feelings of social responsibility tended to influence their considerations. Becoming diagnosed could be a frightening prospect. Their living conditions, or the living conditions of others, could be at risk as a result of the diagnosis. One man, who was temporarily working in horticulture and supporting his family by sending them money, learned that others from his home country who had been diagnosed with TB had been forced to give up their jobs. So, although he suspected that he might have TB, he said, “I had to go on working as long as possible. The pain in my chest kept getting worse, but I went on working until my employer said that I had to go and see a doctor” (observation day 2). Hearing stories about others, who were diagnosed with TB, without actually having known the person involved, also influenced the decision to seek medical advice. An example is the story about whether someone who was diagnosed could still sell the newspaper for the homeless. Selling this paper is often seen as the only way for homeless people to earn a legitimate salary. One man said “I hear you are sent home and cannot come back until the treatment is over. Then how would I support myself?” (interview 3).

At the same time, there was a social pressure to do the right thing. The risk of having a disease that could infect other people was not acceptable. This was not just a personal choice; others emphasized that it was socially unacceptable to refuse to take a medical examination. This was observed when walking around the inner city, looking for people at risk of TB and offering them easy access to free sputum tests. A group of people sitting on a bench drinking encouraged each other to participate. As one young man said to another who was undecided on whether or not to take the test, because he was so scared that he might be infected and just wanted it to go away: “You have a responsibility to take the examination. If you are infected it is not just you that is affected. You shouldn't put other people at risk” (observation day 6).

The disease, being potentially life-threatening, seemed to strengthen the pressure to do the right thing. One way of building up this pressure between people was to talk about others that had TB but had not sought medical help in time. During observations among the same group of men, the story of a man who died of TB was told over and over again, each time a new person joined the group.

### Being publicly diagnosed

Undergoing the medical examination and being diagnosed with TB meant having a public diagnosis. Having an infectious, contagious, and potentially life-threatening disease put strong pressure on the patients to contact and provide the names of everyone with whom they had had physically close interaction. The patients did not have the choice of keeping the diagnosis private towards their family, friends, or healthcare professionals. They had to talk about it, and to explain to everyone about the disease and its treatment. This involved not only people with whom they had a more or less close relationship, but sometimes also people with whom they had had only a brief interaction. Subsequently, those people would talk about the diagnosis to even more people. This demand for openness and public awareness of the diagnosis continued throughout the treatment.

It was challenging for the patients to have to talk about the disease to people who would then have to undergo a medical examination to see whether or not they had been infected. The patients would need to know about the symptoms and treatment, because the persons they informed about the risk of infection immediately wanted to know how they could tell whether they were ill, and what symptoms to watch out for. The risk of spreading the infection among other healthy people was limited seen from the healthcare professionals’ point of view, and they tried to pass this knowledge on to the patients. The patients, on the other hand, found it difficult to pass this knowledge on. One patient said: “If I say to someone you might be ill and you need to go to the hospital to get checked, of course they get scared” (interview 1). Some people tried to hide the diagnosis for as long as possible. One young man produced a list of names of the people he lived together with at a youth centre. He said: “I haven't told them I have TB, I said it was pneumonia. When you call them, can you just say that they need to have an X-ray to see whether they have pneumonia as well?” (observation day 5). When the nurse replied that she could not lie to the people she would have to call, he just nodded, already knowing that this was not an option.

### Undergoing treatment

Treatment involved taking various medications at the same time every day, which implied that the patient had to live a regular life during treatment. This was challenging. One man who at the time of diagnosis had been unemployed for several years was offered a job at a garage shortly after initiating treatment. At the interview with the healthcare professionals, he said:

I had to turn it down. I can't cope with having to take medication at the same time every day, and fit that in with getting up and going to work somewhere where I would have to plan according to their time schedule. (Interview 2)

Because the diagnosis was public, the social interactions of the patients changed. The relationship between the healthcare professional and the patient focused on treatment and compliance to treatment. DOT was a common way of helping patients who needed assistance to administer and remember to take their medication. DOT could be carried out either in the patients’ own home by a district nurse, or at a public nursing centre. Nursing care involved more than just helping the patient to take the right medication; the nurses also observed and questioned the patient about, for example, weight loss, whether they had a place to sleep, and whether they had other medical problems. The patients accepted this, but this was sometimes also seen as a form of social control. Participant observation field notes exemplify this:

At the outpatient clinic, the man had to stand on scales to see whether he was still losing weight. When this proved to be the case, he said that his refrigerator was not working, and that he therefore ate only bread. The nurse phoned the district nurse to ask whether they could help to bring food to him. While she was away, the patient spoke to me and said: “I am in the middle of sorting out my belongings in my apartment. I have it under control.” The nurse came back and said that the district nurse had said that the apartment was so messy that the electrician could not get into the kitchen to fix the refrigerator. She said that the patient would be hospitalized for a few days to ensure he got proper food, and then the district nurse would arrange for some people to come and help him clean up the apartment so that the refrigerator could be fixed. The patient reluctantly accepted. (Observation day 8)

Healthcare professionals and social workers not acquainted with the disease wanted more protection for themselves and their families. They were afraid that they might bring the disease home to their own families. At a centre where homeless people could stay while they got their lives back together again, the social workers wanted all patients who were coughing to wear masks. They were so worried about being infected that they wanted to clearly identify people who were at risk of TB. At a meeting they discussed whether they could make all occupants wear masks if they coughed. “All of them might be infected” (observation day 2), someone said during the discussion.

Interactions with family and friends were also affected. Close relationships between patients and others were, however, less affected: close friends would remain close friends, even though there was a risk of transmitting the disease. As one man said, “You really get to know who your true friends are” (interview 12). The patients were often asked about who they thought might have infected them, and who they might themselves have infected. An older woman described the time she was given the diagnosis and had called her daughter: “She just said, ‘What about us?’ – without even asking how I was” (interview 13). Despite the reassurance of the healthcare professionals that the risk of transmitting the disease from one person to another was almost eliminated after 2 weeks of medical treatment, this was a message that could sometimes be hard for the patient to get across. One man who had been in treatment for a while talked about how his social life had changed: “Before, I could go into a bar and wave a 50 kroner note, and I would have 10 friends. Now I can wave a 100 kroner note and I have no friends” (interview 12). He did not expect his social life to return to normal for a very long time, because he recognized this pattern from the time when his mother was treated for TB some years previously.

In striving to regain a normal life as it was before the diagnosis, the patients struggled to regain control. Regaining control sometimes meant forgetting about the disease and the treatment for a while, and going back to life as it was before. For some patients, this was emphasized by their abuse. As one nurse said: “He has missed an appointment, but there is no sense in phoning him today. It has just been his payday, and I expect he is out there somewhere spending it on beer. I'll phone him in a day or two, when he is sobering up” (observation day 8).

For others, the diagnosis served as a reminder of things that the patient had been thinking about for a while, but had not had the strength to change. Living a life with substance abuse, not taking care to get sufficient nutrition, being homeless, or finding oneself in some other kind of difficult social situation meant living with the risk of becoming ill as a result of that lifestyle. The diagnosis brought about a need to consider how to regain control by changing one's living conditions in order not to become even more ill. One man explained his plans: “I have been a bit loose, but when I am well again I will stop smoking hash, get an apartment and a job. I am getting too old for this kind of life” (interview patient 14).

The concern for others and the need to regain control over what happened to them continued for a long time. There was a feeling of needing to protect others: not only close relations, but anyone who had been at risk of infection, as a result of possibly having been responsible for spreading disease.

Sometimes this even meant having to be responsible for sorting out differing kinds of medical advice. In one case a family of three were all diagnosed with TB, as a result of which a relatively high number of people with whom they had interacted with were recommended to undergo medical checks. When asked what he felt was most challenging at the present time, the father said:

Everyone keeps calling me. Our friends, colleagues and families live all over the country. They have visited at least five different hospitals, and all of them have received different advice. Some were told to get a Mantoux (skin test), some were told to come only if they had symptoms, some will be monitored with chest X-rays for a year, and so on. It is like no-one can tell me what is right or wrong. Does no-one know? Is there anyone I can talk to who can tell me the right way to make sure that a person is not infected? (Interview 9)

## Discussion

In this article we have identified a main concern among patients diagnosed with TB, which is “being publicly diagnosed.” Confidentiality over health issues between healthcare professionals and patients is based on ethical and legal rules, and is internationally affirmed in the WHO Patients’ Charter for Tuberculosis Care (WHO, 2006). This confidentiality is challenged by asking patients to report any person with whom they have had close contact, because these people are then contacted and offered clinical assessment. These contacts are then informed of the TB diagnosis of the index patient, and they are not subject to the same rules of confidentiality. Even though health care professionals ask for the patients’ consent before contacting other potentially infected persons, confidentiality about the diagnosis is usually impossible to sustain. In our study we found that feelings of social responsibility influenced considerations of biding one's time or deciding to undergo treatment, and thereby the decision to seek out healthcare professionals. This was also found in a Swedish study, in which routine contact tracing was a special concern among immigrants, because they feared confidentiality would be broken and their medical history shared with the immigration authorities (Kulane, Ahlberg, & Berggren, 2010).

Issues of confidentiality and self-determination in relation to telling other people about one's disease have been found to be of importance in various cultures (Eastwood & Hill, 2004) and as a basis for implementing voluntary testing in relation to other infectious diseases, such as HIV (Sauka & Lie, 2000).

Previous studies into the decisions of TB patients on when to seek medical advice have interpreted the period from experiencing bodily symptoms to seeking health care as a period of symptom misinterpretation (Nnoaham, Pool, Bothamley, & Grant, 2006). In other studies relating to other diseases, the interpretation of the physical symptoms has been found to be influenced not only by the ability to interpret a symptom, but also by the person's social situation, biography, and life expectations (Andersen, Paarup, Vedsted, Bro, & Soendergaard, 2010). The influence of social conditions was also found in our study, and is supported by a study from another low-incidence country in which patients, before undergoing examination and seeking treatment, were found to first consider such factors as their financial situation, the severity of symptoms, and the fear of being labelled or stigmatized (Sagbakken, Bjune, & Frich, 2010).

The stigma that has been identified with having TB has usually been studied as a problem for the control of the disease, rather than from the perspective of the patient (Macq, Solis, & Martinez, 2006). One review identified the stigma as having a negative impact on patients and their families, with the possible consequence of them withdrawing from social interaction (Juniarti & Evans, 2011). In our study the view of changed social interactions is dual, because it was determined both by the patients’ withdrawal from social interaction, and by the withdrawal of social interaction from the patient. In our study, this withdrawal was even found to influence the patients’ decision of whether or not to undergo an examination. Limited research has been done on how to intervene to increase patients’ empowerment in relation to TB, but the sense of empowerment seems to be highly context-dependent (Macq, Torfoss, & Getahun, 2007), and may also be related to the language used by the healthcare professional (Zachariah et al., 2012) or the patient's interpretation of the support-givers’ motives (Ushie & Jegede, 2012). The finding in this study of the efforts to restore control might be seen as an element of empowerment. Helping patients to regain control might therefore be a successful element in regaining empowerment. Additional research will, however, be needed.

The patients’ efforts to regain control sometimes meant that they chose actions that counteracted treatment compliance, seen from a medical perspective. The experience of treatment as encompassing social control may be related to the experience of humiliation or eroded integrity which has been identified as a possible consequence of DOT (Sagbakken, Bjune, & Frich, 2012; Sagbakken, Frich, Bjune, & Porter, 2013). It has been suggested that better professional–patient interaction during DOT may improve adherence as well as respecting the patient's integrity (Mishra, Hansen, Sabroe, & Kafle, 2006).

The data used in this study represents experiences from patients who were in contact with healthcare professionals in two specialized pulmonary medical wards. It is thus possible that the experience of patients examined and diagnosed in different medical specialties is not covered in this study. A number of patients did not understand or speak Danish; the voices, experiences, and cultural context of these patients are missing, and might have offered additional understanding.

## Conclusion and implications

The findings of this study offer new insight into the experiences of patients diagnosed with TB in a Danish, high-income, low-TB-incidence setting. The findings also offer an empirically derived basis for developing interventions aimed at reducing the time from infection or the appearance of symptoms to medical examination, and increasing the wellbeing of the patients. Suggestions for elements of future interventions include organizing a patient trajectory where confidentiality is a main focus, increasing public awareness of the disease and decreasing the burden involved in being diagnosed, collaborative national development and implementation of clinical guidelines for the examination and treatment of TB, and increasing the possibilities for individualized social and medical support of patients.

## References

[CIT0001] Alvarez J. L, Kunst A. E, Leinsalu M, Bopp M, Strand B. H, Menvielle G (2011). Educational inequalities in tuberculosis mortality in sixteen European populations. International Journal of Tuberculosis and Lung Disease.

[CIT0002] Andersen R. S, Paarup B, Vedsted P, Bro F, Soendergaard J (2010). ‘Containment’ as an analytical framework for understanding patient delay: A qualitative study of cancer patients’ symptom interpretation processes. Social Science and Medicine.

[CIT0003] Dara M, De Colombani P, Petrova-Benedict R, Centis R, Zellweger J. P, Sandgren A (2012). Minimum package for cross-border TB control and care in the WHO European region: A Wolfheze consensus statement. European Respiratory Journal.

[CIT0004] Eastwood S. V, Hill P. C (2004). A gender-focused qualitative study of barriers to accessing tuberculosis treatment in The Gambia, West Africa. International Journal of Tuberculosis and Lung Disease.

[CIT0005] Gandy M, Zumla A (2002). The resurgence of disease: Social and historical perspectives on the ‘new’ tuberculosis. Social Science and Medicine.

[CIT0006] Glaser B. G (1978). Theoretical sensitivity: Advances in the methodology of grounded theory.

[CIT0007] Glaser B. G (1992). Basis of grounded theory analysis: Emergence vs. forcing.

[CIT0008] Glaser B. G, Strauss A. L (1968). The discovery of grounded theory: Strategies for qualitative research.

[CIT0009] Juniarti N, Evans D (2011). A qualitative review: The stigma of tuberculosis. Journal of Clinical Nursing.

[CIT0010] Kamper-Jorgensen Z, Andersen A. B, Kok-Jensen A, Kamper-Jorgensen M, Bygbjerg I. C, Andersen P. H (2012). Migrant tuberculosis: The extent of transmission in a low burden country. BMC Infectious Diseases.

[CIT0011] Kulane A, Ahlberg B. M, Berggren I (2010). “It is more than the issue of taking tablets”: The interplay between migration policies and TB control in Sweden. Health Policy.

[CIT0012] Lillebaek T, Andersen A. B, Seersholm N. J, Thomsen V. O (2012). [Continued problems with tuberculosis among Danes and Greenlanders in Denmark and the need for reinforced control—A systematic review]. Ugeskrift for Laeger.

[CIT0013] Lomborg K, Kirkevold M (2003). Truth and validity in grounded theory—a reconsidered realist interpretation of the criteria: Fit, work, relevance and modifiability. Nursing Philosophy.

[CIT0014] Macq J, Solis A, Martinez G (2006). Assessing the stigma of tuberculosis. Psychology Health & Medicine.

[CIT0015] Macq J, Torfoss T, Getahun H (2007). Patient empowerment in tuberculosis control: Reflecting on past documented experiences. Tropical Medicine and International Health.

[CIT0016] Mishra P, Hansen E. H, Sabroe S, Kafle K. K (2006). Adherence is associated with the quality of professional-patient interaction in Directly Observed Treatment Short-course, DOTS. Patient Education and Counseling.

[CIT0017] Munro S. A, Lewin S. A, Smith H. J, Engel M. E, Fretheim A, Volmink J (2007). Patient adherence to tuberculosis treatment: A systematic review of qualitative research. PLoS Medicine.

[CIT0018] National Collaborating Center for Chronic Conditions (2006). Tuberculosis: Clinical diagnosis and management of tuberculosis, and measures for its prevention and control.

[CIT0019] Nnoaham K. E, Pool R, Bothamley G, Grant A. D (2006). Perceptions and experiences of tuberculosis among African patients attending a tuberculosis clinic in London. International Journal of Tuberculosis and Lung Disease.

[CIT0020] Oeye C, Bjelland A. K, Skorpen A (2007). Doing participant observation in a psychiatric hospital—Research ethics resumed. Social Science and Medicine.

[CIT0021] Pozsik C. J (1993). Compliance with tuberculosis therapy. Medical Clinics of North America.

[CIT0022] Raviglione M. C, Snider D. E, Kochi A (1995). Global epidemiology of tuberculosis. Morbidity and mortality of a worldwide epidemic. JAMA.

[CIT0023] Sagbakken M, Bjune G. A, Frich J. C (2010). Experiences of being diagnosed with tuberculosis among immigrants in Norway—factors associated with diagnostic delay: A qualitative study. Scandinavian Journal of Public Health.

[CIT0024] Sagbakken M, Bjune G. A, Frich J. C (2012). Humiliation or care? A qualitative study of patients’ and health professionals’ experiences with tuberculosis treatment in Norway. Scandinavian Journal of Caring Science.

[CIT0025] Sagbakken M, Frich J. C, Bjune G. A, Porter J. D (2013). Ethical aspects of directly observed treatment for tuberculosis: A cross-cultural comparison. BMC Medical Ethics.

[CIT0026] Sauka M, Lie G. T (2000). Confidentiality and disclosure of HIV infection: HIV-positive persons’ experience with HIV testing and coping with HIV infection in Latvia. AIDS Care.

[CIT0027] Ushie B. A, Jegede A. S (2012). The paradox of family support: Concerns of tuberculosis-infected HIV patients about involving family and friends in their treatment. AIDS Patient Care STDS.

[CIT0028] Volmink J, Garner P (2007). Directly observed therapy for treating tuberculosis. The Cochrane Database of Systematic Review.

[CIT0029] WHO (2006). The patients’ charter for tuberculosis care. Patients’ rights and responsibilities World Care Counsil.

[CIT0030] WHO (2010). Treatment of tuberculosis. Guidelines.

[CIT0031] WHO (2012). Global tuberculosis Report 2012.

[CIT0032] Zachariah R, Harries A. D, Srinath S, Ram S, Viney K, Singogo E (2012). Language in tuberculosis services: Can we change to patient-centred terminology and stop the paradigm of blaming the patients?. International Journal of Tuberculosis and Lung Disease.

